# LncRNA *MIR22HG* abrogation inhibits proliferation and induces apoptosis in esophageal adenocarcinoma cells via activation of the STAT3/c-Myc/FAK signaling

**DOI:** 10.18632/aging.102071

**Published:** 2019-07-10

**Authors:** Wenmei Su, Chunfang Guo, Lihui Wang, Zhuwen Wang, Xia Yang, Feiyu Niu, Daniel Tzou, Xiao Yang, Xiaobi Huang, Jiancong Wu, Xiaorao Chen, Lei Zou, Zhixiong Yang, Guoan Chen

**Affiliations:** 1 Department of Oncology, Affiliated Hospital of Guangdong Medical University, Zhanjiang, China; 2 Department of Surgery, University of Michigan, Ann Arbor, MI 48109, USA; 3 Key Laboratory of Longevity and Aging-related Diseases of Chinese Ministry of Education, Center for Translational Medicine and School of Preclinical Medicine, Guangxi Medical University, Nanning, China; 4 Department of Respiratory and Critical Care Medicine, The Second Affiliated Hospital of Xian Jiaotong University, Xi’an, China; 5 Affiliated Cancer Hospital and Institute of Guangzhou Medical University, Guangzhou, China; 6 Department of Organ Transplant, First Affiliated Hospital of Kunming Medical University, Kunming, China; 7 School of Medicine, Southern University of Science and Technology, Shenzhen, China

**Keywords:** esophageal adenocarcinoma, *MIR22HG*, proliferation, migration, invasion

## Abstract

Long non-coding RNAs (lncRNAs) have involved in human malignancies and played an important role in gene regulations. The dysregulation of lncRNA *MIR22HG* has been reported in several cancers. However, the role of *MIR22HG* in esophageal adenocarcinoma (EAC) is poorly understood. Loss of function approaches were used to investigate the biological role of *MIR22HG* in EAC cells. The effects of *MIR22HG* on cell proliferation were evaluated by WST-1 and colony formation assays. The effects of *MIR22HG* on cell migration and invasion were examined using transwell assays. QRT-PCR and Western blot were used to evaluate the mRNA and protein expression of related genes. In this study, abrogation of *MIR22HG* inhibited cell proliferation, colony formation, invasion and migration in EAC 3 cell lines (OE33, OE19 and FLO-1). Mechanistically, *MIR22HG* silencing decreased the expression of STAT3/c-Myc/p-FAK proteins and induced apoptosis in EAC cell lines. These results delineate a novel mechanism of *MIR22HG* in EAC, and may provide potential targets by developing lncRNA-based therapies for EAC.

## INTRODUCTION

Esophageal adenocarcinoma (EAC) is one of the most aggressive malignancies with poor patient survival worldwide. Much progress has been made in the molecular understanding of EAC, including tumor suppressor gene mutations, aberrant protein expression and cancer stem cell identification. However, the precise molecular mechanism involved in EAC remains unclear. Thus, understanding additional carcinogenesis mechanisms of EAC is urgently needed for developing new therapies for clinical application.

Noncoding RNAs (ncRNAs), including miRNA, circRNA, and long noncoding RNA (lncRNA), account for more than 90 % of the human genome, while protein-coding genes account for less than 2 % of human genome [1]. LncRNAs, which are 200-10,000 nucleotide, control gene expression at epigenetic, transcriptional and post-transcriptional levels. It has been proven that lncRNAs can positively or negatively affect the coding gene expression by multiple mechanisms, such as chromatin remodeling, transcriptional interference, modulating alternatively splicing patterns, as well as many other mechanisms [2]. A number of studies have shown that ncRNAs are capable of influencing various cellular processes such as cell proliferation, cell cycle progression, cell growth, and apoptosis [3–6], and their misexpression confers tumor initiation, cancer cells growth and metastasis [7–9]. Thus, lncRNAs are linked with carcinogenesis and provide a new pathway in cancer research. In recent years, several lncRNAs, including taurine upregulated gene 1 (TUG1) [10], second chromosome locus associated with prostate-1(SChLAP1) [11], colorectal neoplasia differentially expressed (CRNDE) [12], and castration-resistant prostate cancer (CRPC) [13], have been reported to regulate tumor cell growth and progression by altering the balance between cell proliferation and apoptosis. LncRNAs also play essential roles in human malignancies and function as tumor suppressors or oncogenes [14–17]. Collectively, the results suggest that clinical-oriented research on lncRNAs in EAC should be undertaken and further research studies should be designed to discover more tumor-related lncRNAs as candidates of prognostic biomarkers and therapeutic targets.

Recently, some reports show that one lncRNA *MIR22HG* suppresses the cell progression in several cancer, such as cholangiocarcinoma [18], hepatocellular carcinoma [19], gastric cancer [20], and lung cancer [21]. However, the role of *MIR22HG* and the underlying molecular mechanism in the development of EAC remains to be unexplored. In this study, we performed the functional and mechanistic study on lncRNA *MIR22HG* on EAC cells including OE19, OE33 and FLO1. We found that the proliferation, colony formation, migration and invasion were decreased after Knockdown of *MIR22HG* on EAC cell lines. The STAT3, c-Myc and p-FAK proteins were decreased upon *MIR22HG* abrogation. Thus, *MIR22HG* could potentially function as an oncogenic gene in EAC and may provide a potential therapeutic target in EAC.

## RESULTS

### Knockdown of *MIR22HG* suppresses cell proliferation in EAC cells

**Figure 1:**
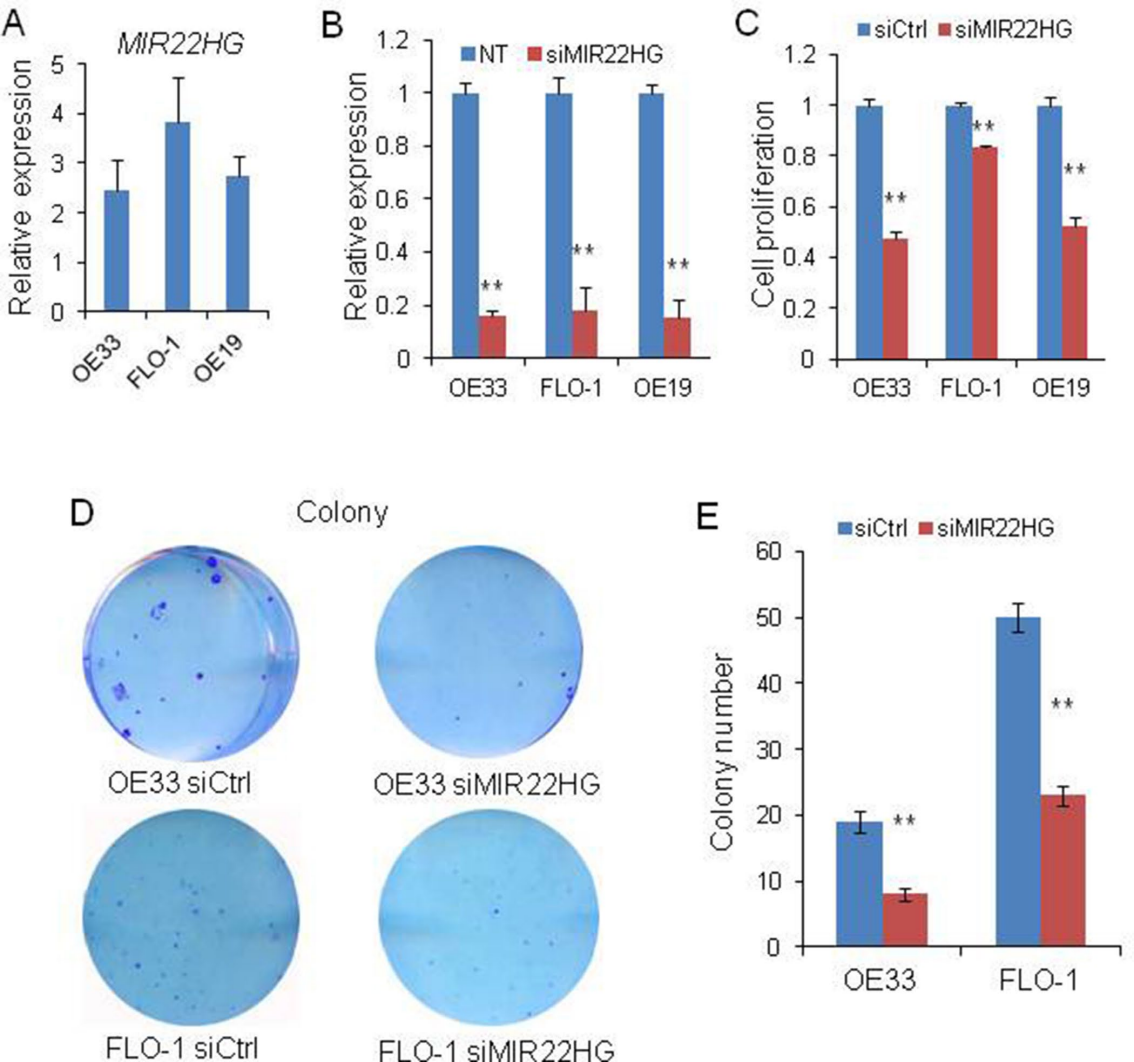
**Effects of knockdown of *MIR22HG* on EAC cells viability.** (**A**) relative expression of *MIR22HG* in OE33, FLO-1 and OE19 cell lines. (**B**) The *MIR22HG* expression level indicating the knockdown efficiency of siRNA determined by qRT-PCR in 3 EAC cells transfected with *siMIR22HG*. (**C**) WST-1 assays were used to determine the cell viability after *MIR22HG* knockdown with siRNA in OE33, FLO-1 and OE19 cells. (**D**), Colony formation in OE33 and FLO1 cells after *MIR22HG* knockdown. (**E**) Bar chart counting the number of colonies from Figure 1D. Values represented the mean ± s.d. from three independent experiments. **P < 0.01.

To evaluate the biological roles of *MIR22HG* on EAC, we first tested the *MIR22HG* expression in OE33, FLO-1 and OE19 cell lines and found this gene was expressed in these cells (Figure 1A). and then we performed *MIR22HG* knockdown with siRNA in these 3 EAC cell lines. QRT-PCR assays indicated that *MIR22HG* expression was significantly reduced more than 80% after transfection with *MIR22HG* siRNA (Figure 1B). Functionally, we found that the cell proliferation measured by WST-1 assays was significantly decreased upon knockdown of *MIR22HG* in OE33, FLO-1 and OE19 cells (Figure 1C). In consistent with WST -1 assay results, knockdown of *MIR22HG* significantly inhibited the colony formation ability of the EAC cells compared with the non-target control (Figure 1D and 1E). These results suggested that *MIR22HG* may play an oncogenic role in regulating EAC cell growth.

### Knockdown of *MIR22HG* inhibits EAC cell migration and invasion

**Figure 2:**
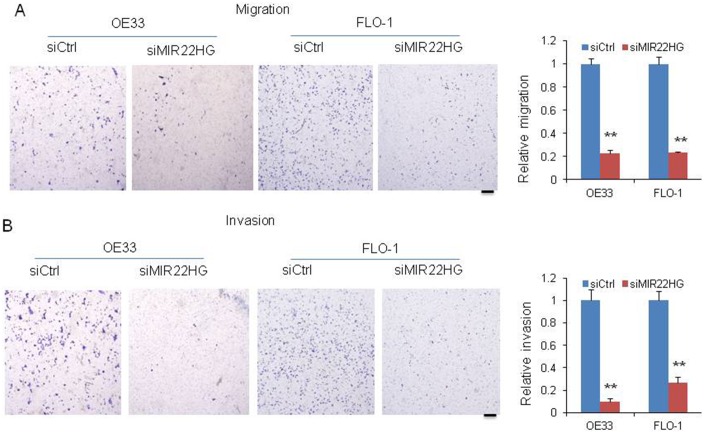
**Knockdown of *MIR22HG* inhibits cancer cell migration and invasion.** Migration (**A**) and invasion (**B**) were decreased after *MIR22HG* siRNA transfection in OE33 and FLO-1 cells. The bar chart shows the relative number of migration and invasion cells. Scale bar: 5mm. Values represented the mean ± s.d. from three independent experiments. *P < 0.05, **P < 0.01.

To further determine whether *MIR22HG* is involved in the cell migration and invasion, we performed matrigel-coated transwell experiments. We observed that knockdown of *MIR22HG* significantly decreased the migration and invasion potential in OE33 and FLO-1 cells (Figure 2A and 2B), indicating that *MIR22HG* may have a role in EAC metastasis or tumor progression.

### *MIR22HG* abrogation inhibits STAT3, c-Myc and p-FAK proteins expression and induces apoptosis

**Figure 3:**
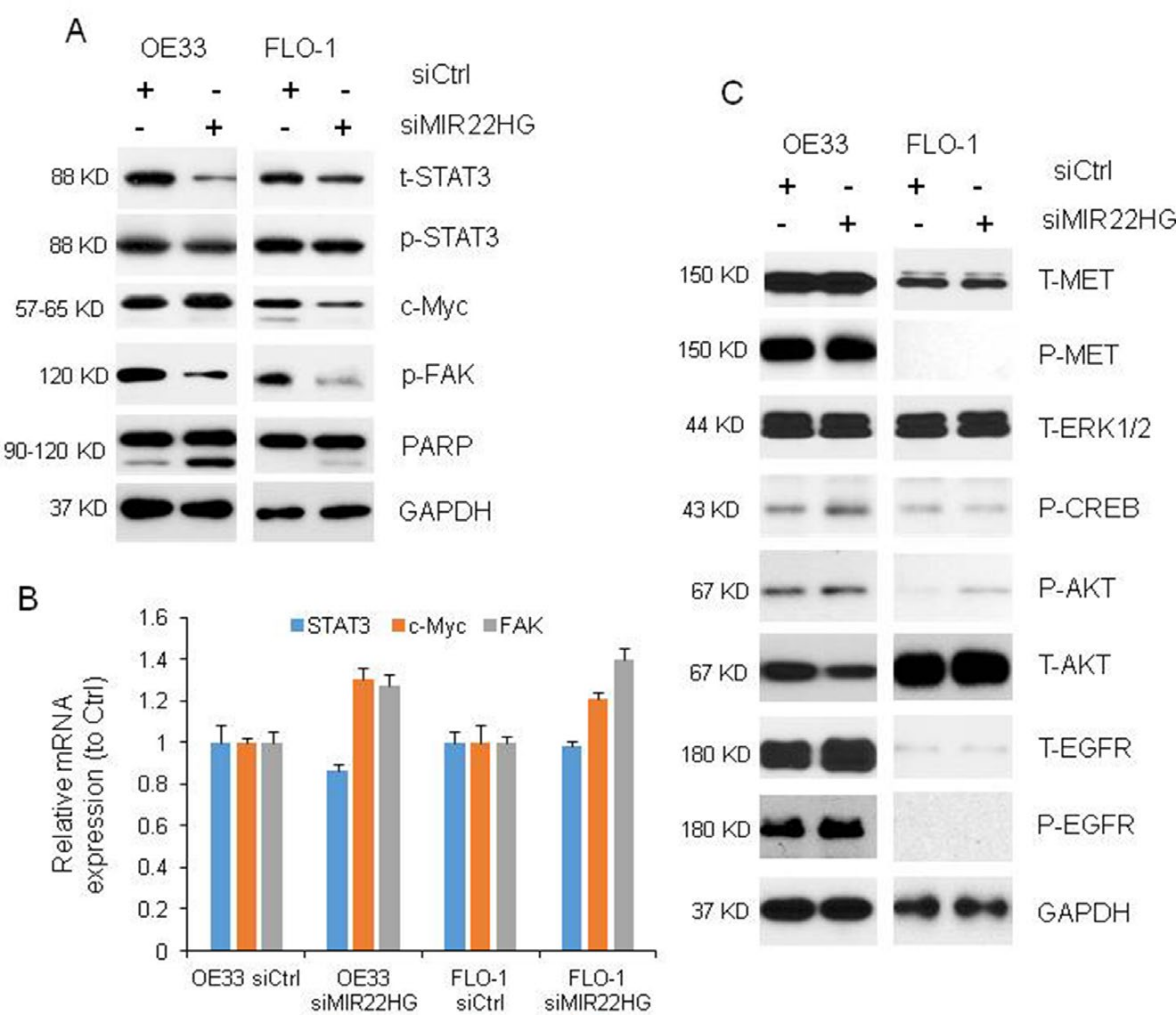
**Proteins and mRNAs regulated by knockdown of *MIR22HG*.** (**A**) Protein levels of t-STAT3, p-STAT3 and p-FAK were regulated by *MIR22HG* siRNA in OE33 and FLO cells, and c-Myc was changed in FLO1 cells by Western blotting. PARP cleavage was also induced by *MIR22HG* siRNA in OE33 and FLO1 cells. GAPDH was used as a protein loading control. (**B**) qRT-PCR showing the mRNA expressions of STAT3, c-MYC and FAK in OE33 and FLO1 cells. GAPDH was used as control. (**C**) MET, EGFR, AKT and ERK1/2 proteins were not changed after *MIR22HG* siRNA treatment at 72 hours.

To understand the mechanisms of *MIR22HG* roles in regulating EAC cell proliferation in OE33 and FLO-1 cells, we performed western blot and found that knockdown of *MIR22HG* resulted in reduced total and phosphor STAT3 (t-STAT3 and p-STAT3) as well as phosphor FAK (p-FAK) proteins expression in OE33 and FLO-1 cell lines, while c-Myc protein was decreased in FLO-1 cells but unchanged in OE33 cells (Figure 3A). The mRNA levels of STAT3, c-MYC and FAK were not decreased after *MIR22HG* knockdown (Figure 3B) indicating that *MIR22HG* affected STAT3, c-Myc and p-FAK proteins may be at the post-transcriptional level. We did not find that MET, EGFR, AKT and ERK1/2 proteins were changed after *MIR22HG* siRNA treatment at 72 hours (Figure 3C), indicating that MET and EGFR signaling were not involved in *MIR22HG* regulation in EAC.

It has been known that cleavage of PARP (c-PARP) is one of apoptosis marker. We found that the c-PARP was increased after knockdown of *MIR22HG* with siRNA at 72 h (Figure 3A), suggested that *MIR22HG* could regulate both cell proliferation and programs cell death in esophageal cancer cells.

### *MIR22HG* regulates c-Myc/p-FAK and apoptosis via STAT3

**Figure 4:**
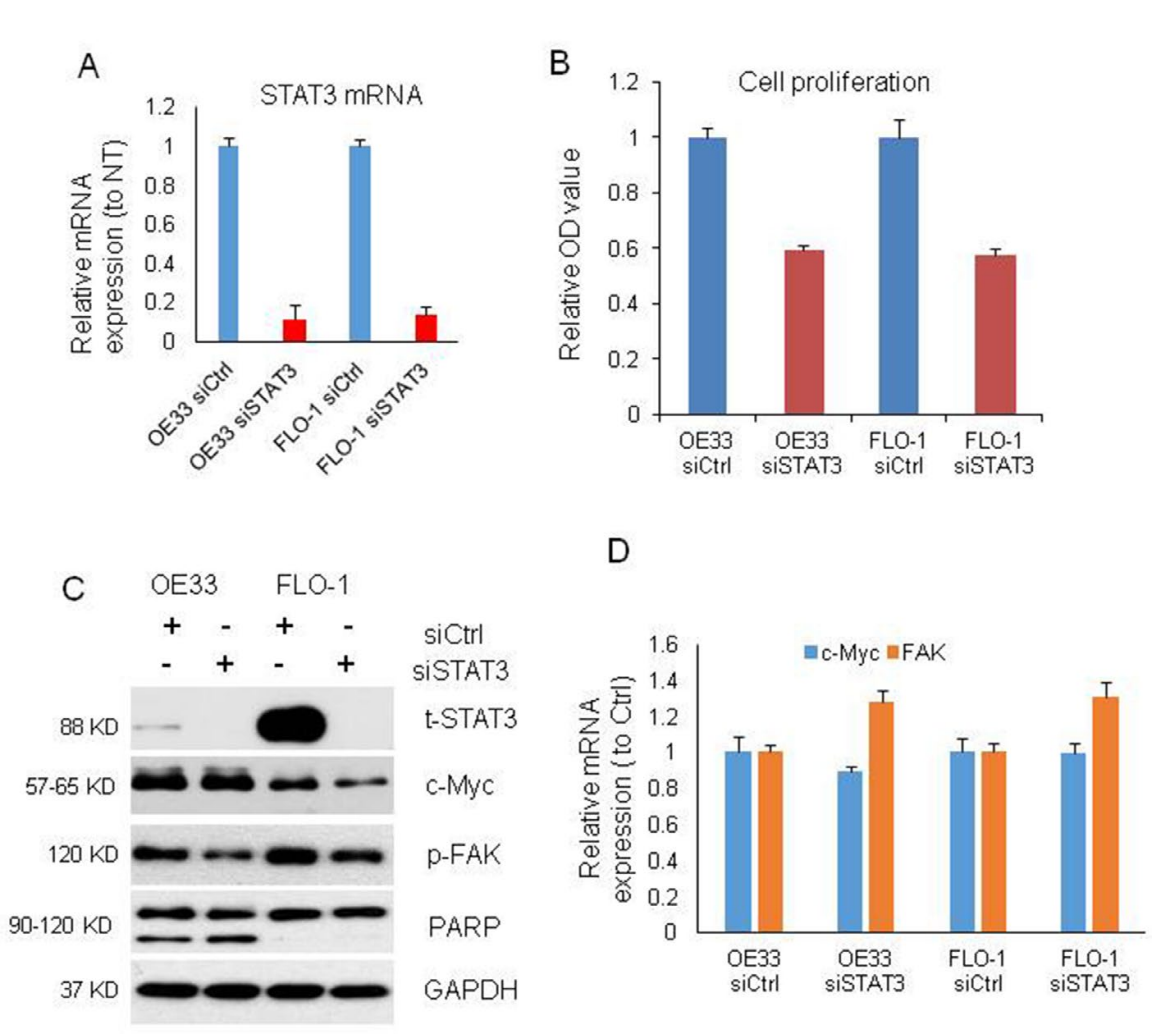
**Cell proliferation, proteins and mRNAs regulated by knockdown of STAT3.** (**A**) STAT3 mRNA expression was decreased by more than 80% after STAT3 knockdown with siRNA on OE33 and FLO1 cells measured by qRT-PCR. (**B**) WST-1 assays were used to determine the cell viability for STAT3 siRNA transfecting OE33 and FLO1 cells. Values represented the mean ± s.d. from three independent experiments. (**C**) Protein levels of t-STAT3 and p-FAK were regulated by STAT3 siRNA in OE33 and FLO cells, and c-Myc was also changed in FLO1 cells by Western blotting. PARP cleavage was also induced by STAT3 siRNA in OE33 cells. GAPDH was used as a protein loading control. (**D**) qRT-PCR showing the mRNA expression of c-MYC and FAK in OE33 and FLO1 cells. GAPDH was used as control. Values represented the mean ± s.d. from three independent experiments. *P < 0.05, **P < 0.01.

**Figure 5:**
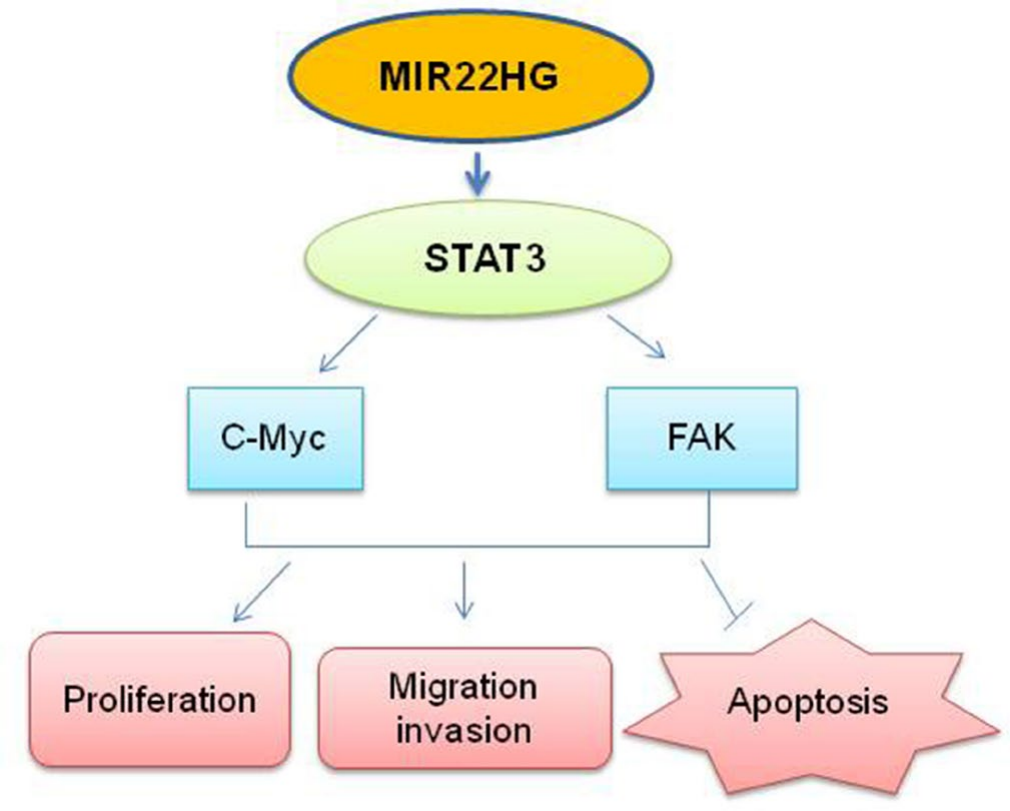
**Schematic the potential signaling affected by knockdown of *MIR22HG*.** *MIR22HG* siRNA inhibits STAT3 proteins, then affects c-MYC and FAK proteins to modulate the cells proliferation, migration, invasion and induced apoptosis in esophagus cancer.

To further make clear the relationship among *MIR22HG*, STAT3, c-Myc and p-FAK proteins and roles in EAC proliferation and apoptosis, we performed knockdown of STAT3 with siRNA in OE33 and FLO-1 cells (Figure 4A). The cell proliferation was decreased by 40% upon STAT3 knockdown in OE33 and FLO-1 cells at 120 h (Figure 4B) and apoptosis was induced in OE33 (Figure 4C). We found that p-FAK protein was decreased in OE33 and FLO1 cells, while c-Myc was decreased in FLO-1 cells and unchanged in OE33 cells (Figure 4C), which was similar as *MIR22HG* knockdown in EAC cell lines (Figure 3A). While the mRNAs of c-Myc and FAK were not changed (Figure 4D). These results suggest that *MIR22HG* mediated control of EAC cell proliferation and apoptosis may occur via the STAT3/c-Myc/p-FAK axis (Figure 5).

**Figure 6:**
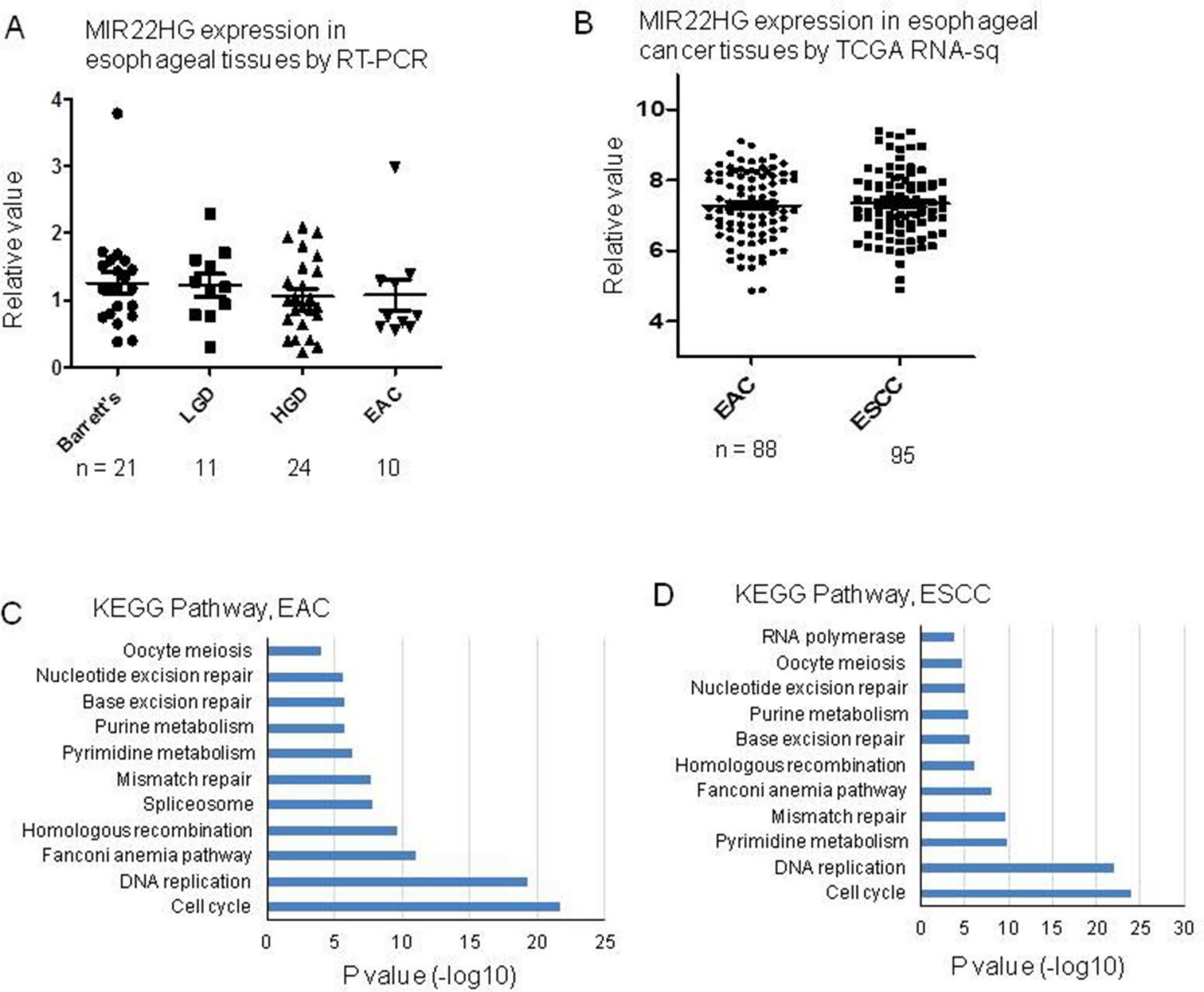
**MIR22HG expression in esophageal tissues and pathway involved by MIR22HG negative correlated genes.** (**A**) MIR22HG expression of esophageal adenocarcinomas (EAC), high grade dysplasia (LDH), low grade dysplasia (LDH) and Barrett’s measured by RT-PCR. There is no significant different among them (p > 0.05). (**B**) MIR22HG expression from TCGA RNA-seq data including 88 esophageal adenocarcinomas (EAC) and 95 esophageal squamous cell carcinomas (ESCC). There is no significant different between EAC vs. ESCC (p > 0.05). (**C** and **D**), DAVID pathway analysis of MIR22HG negative correlated genes indicating that the cell cycle and DNA replication pathways were the most significantly involved pathways in both EAC and ESCC (p < 0.001).

To explore the expression status of *MIR22HG* in primary EAC tumors, we first performed RT-PCR for *MIR22HG* expression from University of Michigan samples including EAC, high grade dysplasia (LDH), low grade dysplasia (LDH) and Barrett’s. There was no significant different among these groups (Figure 6A). We then analyzed *MIR22HG* expression from TCGA RNA-seq data including 88 EAC and 95 esophageal squamous cell carcinomas (ESCC). There was no significantly finding regarding patient survival, stage and EAC vs. ESCC (Figure 6B). We also performed DAVID Gene Ontology/pathway analysis of *MIR22HG* correlated (Pearson correlation) genes based on TCGA data, we found that the cell cycle and DNA replication pathways were the most significantly involved pathways in both EAC and ESCC (Figure 6C, 6D).

## DISCUSSION

In the present study, we identified *MIR22HG* as an oncogenic player and revealed a previously unknown mechanism involving *MIR22HG* in EAC biology. We found that knockdown of endogenous *MIR22HG* expression significantly cell proliferation, migration and migration.

Several subsets of genes that act by either activating oncogenes or silencing tumor suppressor genes precisely regulate tumor development and progression [22]. Recent studies showed oncogenes were usually activated by genetic or epigenetic alterations in cancer cells [23, 24]. Su et al. [21] reported that *MIR22HG* triggered cell survival via MET gene. *MIR22HG* suppressed gastric cancer progression through attenuating NOTCH2 signaling [20] *MIR22HG* repressed cell proliferation, migration and invasion in CCA by negatively regulating the Wnt/β-catenin signaling pathway [18]. Until now, we don’t know which signal pathway genes are involved in EAC. To explore the molecular mechanism through which *MIR22HG* contributes to proliferation in EAC, we investigated potential target proteins involved in proliferation. We identified which genes were differentially expressed upon knockdown of *MIR22HG*, in comparison with untreated cells. The protein levels of t-STAT3, p-STAT3 and p-FAK were down regulated by *MIR22HG* siRNA in OE33 and FLO cells, and c-Myc was also decreased in FLO1 cells. The mRNAs of these genes were not changed suggest that *MIR22HG* affects. STAT3, c-Myc and p-FAK protein at the post-transcriptional level. We didn’t find that MET, EGFR, AKT and ERK1/2 proteins were changed after *MIR22HG* siRNA treatment at 72 hours in EAC.

STAT3 becomes inappropriately and constitutively activated in a high percentage of solid malignancies including melanoma, multiple myeloma, and cancers of the breast, ovary, prostate, head and neck, and pancreas [25]. Hyper activated STAT3 promotes the expression of genes involved in cell proliferation, self-renewal, angiogenesis, inflammation-phenotypes and survival, which collectively contribute to malignant transformation and progression [26, 27]. A recent study showed that overexpression of FAK has been shown to block the caspase-3-mediated apoptosis; conversely, inhibition of FAK leads to apoptosis in cancer cells [28]. Cytoskeletal remodeling is critical for cancer cell migration, therefore indispensable for cancer metastasis. FAK signaling that resulted from ECM-induced integrin clustering is intimately involved in the reorganization of cytoskeleton and cell motility [29, 30]. Numerous numbers of evidence indicate that FAK is predominately involved in the promotion of tumor invasion, implicating that FAK is a potential target for anticancer therapeutics. In the process of cancer invasion, the activation of FAK in cancer cells could transmit numerous downstream signal pathways in regulating a variety of cellular events, including cytoskeletal remodeling and EMT, to control cell fate [31–33]. During the occurrence of EMT, degradation of E-cadherin can promotes cancer invasion by allows the release of cell-cell restriction, which is in accordance with the disruption of adherent junctions [34]. These results are supporting evidence that *MIR22HG* abrogation induced apoptosis and decreased migration and invasion ability may be through the inhibition of FAK signaling.

In summary, we found that knockdown of *MIR22HG* has the effect of suppressing EAC proliferation, cell migration and invasion in vitro by inhibiting STAT3/c-Myc/p-FAK proteins (Figure 5). Further insights into the functional and clinical implications of *MIR22HG* and its targets may help with the treatment of EAC.

## MATERIALS ANS METHODS

### Cell culture

The human EAC-derived cell lines OE19, OE33 and FLO1 were obtained from the American Type Culture Collection. OE19 and OE33 cells were grown in RPMI-1640 medium (Gibco, Carlsbad, CA, USA), FLO1 cells were grown in DMEM medium (Gibco, Carlsbad, CA, USA). All mediums were supplemented with 10% fetal bovine serum (Gibco BRL, Gaithersburg, MD, USA) and were maintained in a 37 °C incubator with a humidified atmosphere containing 5% CO_2_.

### Esophageal tissues

Esophageal tissues including adenocarcinomas (EAC), high grade dysplasia (LDH), low grade dysplasia (LDH) and Barrett’s were collected from patients undergoing cancer surgery during the period from 1994 to 2014 at the University of Michigan Health System. None of the patients included in this study received any preoperative radiation or chemotherapy. Informed consents were provided by the patients, and all experimental protocols were approved by the University of Michigan Institutional Review Board and Ethics Committee. Resected specimens were frozen in liquid nitrogen first and then stored at −80°C until used for RNA isolation.

### RNA extraction and real-time PCR

Total RNA was isolated using Trizol reagent (Invitrogen, Carlsbad, CA, USA). First strand cDNA was generated using the Reverse Transcription System Kit (Applied Biosytems) according to manufacturer instructions. For mRNA and lncRNA analyses, real-time PCR was performed as previously described [35]. Expressions of mRNA and lncRNA were normalized with GAPDH. For miRNA analysis, real-time PCR was performed using Power SYBR Green Master Mix (Life Technology Inc.) and was performed with an ABI StepOne Real-Time PCR System (Applied Biosystems) as done previously [35]. The real-time PCR reactions were performed in triplicate. The relative levels of gene expression were represented using the formula ΔCt = Ct_gene_ − Ct_reference_, and the fold change of gene expression was calculated by the 2^−ΔΔCt^ method.

### siRNA mediated knockdown *MIR22HG* in EAC cells

Transfections were performed using the Lipofectamine iMAX kit (Invitrogen) according to the manufacturer’s instructions. The siRNAs of *MIR22HG* or STAT3 and scrambled siRNA (siCtrl) were purchased from Dharmocom. After 48-72 hours incubation with siRNAs (10 nM), cells were harvested for RNA and protein extraction.

### Cell proliferation assay

The cell proliferation was assessed using WST-1 (Roche) according to manufacturer instructions. Briefly, a total of approximately 1 × 10^3^ EAC cells were plated in 96-well plates, at 96 h after transfection with siRNA, added 10 μl/well of WST-1 solution during the last 1 h of culture, and the cell proliferation curves were plotted using the 450 nm and 630nm absorbance at each time point. All experiments were performed in triplicate.

### Colony formation assay

Two hundred siRNAs treated EAC cells were plated into 6-well plates and incubated in RPMI-1640 and DMEM medium with 10% FBS at 37 °C. Fourteen days later, the cells were fixed and stained with 0.1% crystal violet. The number of colonies was counted, with a colony being defined as greater than 50 cells.

### Basement membrane matrix invasion assays

For invasion assays, cells were treated with the indicated siRNAs. After 48 h transfection, cells were trypsinized, counted with a Coulter counter and diluted to a desired concentration (OE33: 2.5 × 10^4^; FLO1: 2.5 × 10^4^. 0.5 ml cell suspension per well). Cells were seeded onto basement membrane matrix Boyden chambers (8-mm pore size, BD) present in the insert of a 24-well culture plate (Matrigel was purchased from BD Company). 20% FBS was added to the lower chamber as a chemoattractant. After 12-24 h, the non-invading cells and EC matrix were gently removed with a cotton swab. Invasive cells located on the lower side of the chamber were stained with Diff-QuikTM Stain Set (SIEMENS), air dried and photographed.

### Western blot analysis

Total cell lysates were prepared with sample buffer and boiled at 95 °C for 5 min. The samples were transferred to SDS–PAGE at 80 V for 3 h and then transferred to PVDF membranes for another 3 h. After incubation with specific antibodies for STAT3, FAK, PARP, c-Myc, CREB, MET, AKT, ERK1/2 and GAPDH at 4 °C overnight, the membranes then were washed by 1% TBST for three times, incubated with secondary antibodies for 1 h, and the membranes were developed using ECL and exposed to X-ray film.

### Statistical analysis

Data were analyzed using GraphPad Prism 6 (GraphPad software) and R software. All data are continuous variables and follow a normal distribution. The other data such as proliferation were evaluated by unpaired Student’s t-test. A two-tailed p value < 0.05 was considered significant.
